# Supplementary material for: GACELLE: GPU-accelerated tools for model parameter estimation and image reconstruction
Source: ArXiv. 2025 Nov 27:arXiv:2511.22094v1. Preprint. [Version 1] (PMC12676372)
Supplement: 1 [file NIHPP2511.22094V1-supplement-1.pdf]

Supplementary Materials

Table S1: Acquisition parameters of the in vivo data used for Demo #1-3.

Demo	#1 NEXI	#2 AxCaliberSMT		#3 MCR-MWI		
**Scanner**	Connectome 2.0		**Scanner**	Prisma		
**DWI**			**Variable-flip-angle-multi-echo GRE**			
**Diffusion time, *D* (ms)**	[13,21,30]	[13,30]	**Protocol**	**#1**	**#2**	**#3**
**#diff. grad. Dir per shells**	32 (lower SNR) or 64 (high SNR)	32 or 64	**Flip angle (°)**	[5,10,20,50,70]		
***b* (ms/μm²)**	[2.3,3.5,4.8,6.5, 11.3*,17.3**] *D≥21ms; **D=30ms	D_{13}=[0.05,0.35,0.8,1.5, 2.4,3.45,4.75,6]; D_{30}=[0.2,0.95,2.3,4.25, 6.75,9.85,13.5,17.8]	**TR/TE_1/ΔTE(ms)/ #TE**	38/2.2/3.07/ 12	50/2.2/3.07/ 15	55/2.68/3.95/ 13
**TR/TE (ms)**	3600/54		**Data type**	Complex-valued		
**SMS/R_{GRAPPA}/PF**	2/2/0.75		**R_{CAIPI}**	5		
**Res. (mm)**	2 (isotropic)		**Res. (mm)**	1.5 (isotropic)		
**TA (min)**	40 (lower SNR) or 80 (high SNR)	56	**TA (min)**	14	18	20

Correspondence to Kwok-Shing Chan

A. Semi-supervised learning for fast multi-compartment relaxometry myelin water imaging (MCR-MWI)

Methods

In multi-compartment relaxometry for myelin water imaging (MCR-MWI) (Chan and Marques, 2020), variable flip angle data introduce T_1 weighting that helps separate myelin water (MW) from intra-/extra-cellular water (IEW), as these compartments exhibit different T_1 relaxation. Imperfect gradient and RF spoiling preclude a simple analytical steady state, therefore, the compartmental T_1-weighted steady-state signal is modelled with the extended phase graph with exchange (EPG-X) formalism (Malik et al., 2017) rather than a Bloch–McConnell closed-form solution. Direct use of EPG-X within voxelwise nonlinear least squares (NLLS) is computationally demanding; without parallelisation, a single whole-brain dataset at 1.5-mm isotropic resolution requires approximately 250 CPU-hours.

To enable fast, parallel evaluation of the EPG-X steady-state signal within *GACELLE*, we trained an artificial neural network (ANN) surrogate to approximate the compartmental steady-state signal. The trained network is embedded in the MCR-MWI forward model and provides fast evaluations during optimisation across large volumes without subject-specific training, maintaining applicability across a range of acquisition settings.

ANN for EPG-X steady-state T_1 weighted signals

Architecture and inputs

We used a multi-layer perceptron (MLP) with 7 hidden layers [20, 30, 40, 45, 60, 65, 75] and leaky RELU activations (scale factor = 0.01), as illustrated in Figure S1. The network accepts 6 inputs: myelin volume fraction f_M, intra-/extra-axonal T_1 ($T_{1,IEW}$),

Correspondence to Kwok-Shing Chan

intra-/extra-axonal T_2 ($T_{2,IEW}$), exchange rate (k_{IEWM}), flip angle (α), and repetition time (TR). Feature transformations were applied to these inputs to produce 11 normalised features. The model contains 15782 learnable parameters. Using the same input format and architecture, two networks were trained: one predicts the differences between the IEW and MW steady-state magnitude signal derived from EPG-X and Bloch-McConnell solution; the other predicts the phase of the IEW steady-state signal, each to match the corresponding EPG-X simulation.

Training data

We generated $2x10^6$ random parameter sets θ with the following parameter ranges: $f_M \in [0,0.72]$, $T_{1,IEW} \in [500,6000]$ ms, $T_{2,IEW} \in [25,4000]$ ms, $k_{IEWM} \in [0,10]$ s^{-1}, TR ∈ [25, 85] ms, $\alpha \in [1,90]$°. Myelin T_1 and T_2 were fixed to 234 ms and 15 ms, respectively. The RF spoiling phase was set to 50°. For each θ, steady-state signals were simulated for α from 1° to 90° (step size = 2.3°).

Training procedures and loss

Networks were trained for 100 epochs using the Adam optimiser. The total loss was the sum of three L1 terms:

$$total\ loss = L1_\theta + L1_{\theta,\alpha\in[1-90]^\circ} + \lambda L1_{dS/d\alpha}\ [Eq.S1]$$

with

$$L1_\theta = \|\Delta S_{EPG-X}(\theta) - S_{ANN}(\theta)\|_1\ [Eq.S2a]$$

$$L1_{\theta,\alpha\in[1-90]^\circ} = \left\|\Delta S_{EPG-X_{\alpha\in[1-90]^\circ}}(\theta,\alpha) - S_{ANN_{\alpha\in[1-90]^\circ}}(\theta,\alpha)\right\|_1, \text{aggregated over } \alpha \in [1-90]^\circ\ [Eq.S2b]$$

$$L1_{dS/d\alpha} = \left\|\frac{d\,\Delta S_{EPG-X_{\alpha\in[1-90]^\circ}}(\theta)}{d\alpha} - \frac{d\,S_{ANN_{\alpha\in[1-90]^\circ}}(\theta)}{d\alpha}\right\|_1, \text{aggregated over } \alpha \in [1-90]^\circ\ [Eq.S2c]$$

where ΔS_{EPG-X} is the signal difference between EPG-X steady-state and Bloch-McConnell solution. The first term enforces fidelity at each θ; the second matches the full flip angle response; the third term aligns the first derivative with respect to α, encouraging smooth, physically plausible steady-state curves. The hyperparameter λ

Correspondence to Kwok-Shing Chan

was initialised at 100 and reduced adaptively across training epochs as $\lambda_i = 100/(1 + 0.1(i - 1))$ where i is the epoch number. The network parameters were trained with a hybrid strategy of increasing batch size and reducing learning rate as a function of the training iterations to ensure convergence.

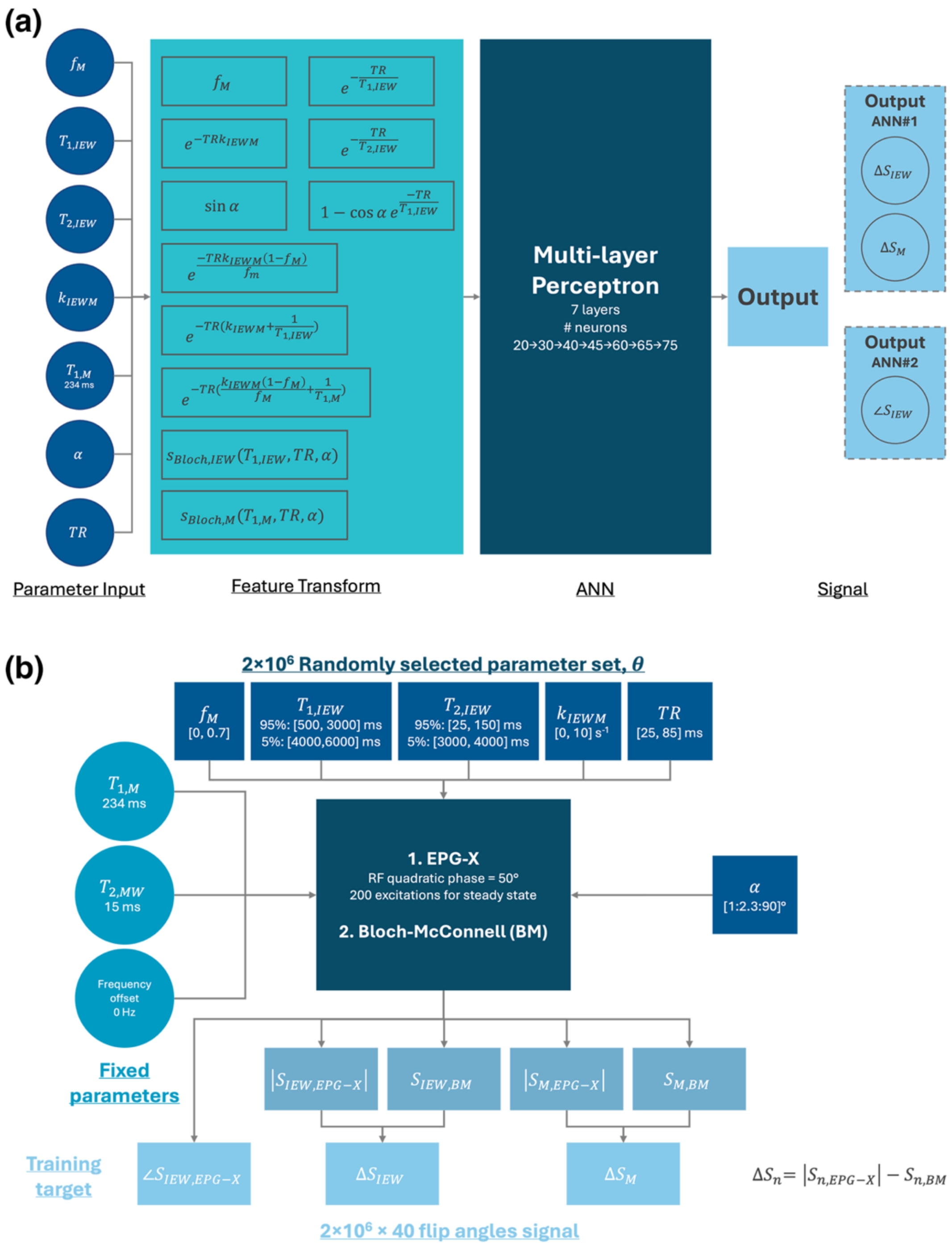

Figure S1: a) The EPG-X steady-state ANN is preceded by a feature transformation step to derive 11 input features that are both normalised to [0,1] and are related to different terms of the Bloch-McConnell

Correspondence to Kwok-Shing Chan

equations. The network core comprises 7 hidden fully-connected layers and a leaky RELU (scale factor=0.01) function. The network parameters are trained with a hybrid strategy of increasing batch size and reducing learning rate as a function of the training iterations to ensure convergence. Note that the first ANN was trained to learn the difference between the magnitude EPG-X and Bloch-McConnell signals, while the second ANN was trained to learn the phase of the IEW signal. b) Illustration of the parameters and their ranges to generate training and validation data.

Integration into *GACELLE*

After training, the ANN replaces the EPG-X computation block within the MCR-MWI forward model used by askadam.m. This preserves model fidelity through the training targets while enabling efficient GPU-accelerated optimisation over entire volumes. No subject-specific retraining is required; users specify acquisition parameters in the forward model, and *GACELLE* performs vectorised evaluation, gradient-based updates, and optional spatial regularisation during fitting.

Validation

To assess the performance of the ANNs, we generated steady-state signals across flip angles from 1° to 90° using three approaches: EPG-X, Bloch-McConnell (BM) equation, and the ANNs, applied to a range of tissue and acquisition parameter sets (Figure S2). In this two-pool model, the BM signal of IEW usually exhibits stronger bias relative to EPG-X, primarily due to prolonged T_2 decay. Across all tested scenarios, the ANN output showed substantially reduced bias compared to BM, indicating that the ANN provides an effective correction for RF spoiling effects. For the phase of the IEW signal, the ANN reproduced the overall trend of the EPG-X simulation, with deviation increasing at higher flip angle and exchange rate; nonetheless, these differences remained within 10% of the true phase values. For the myelin signal, where the short T_2 yields smaller discrepancies between EPG-X and BM, residuals were generally low. Even in this case, ANN correction further reduced the residual bias, demonstrating that the ANN can capture subtle deviations overlooked by the BM formulation.

Correspondence to Kwok-Shing Chan

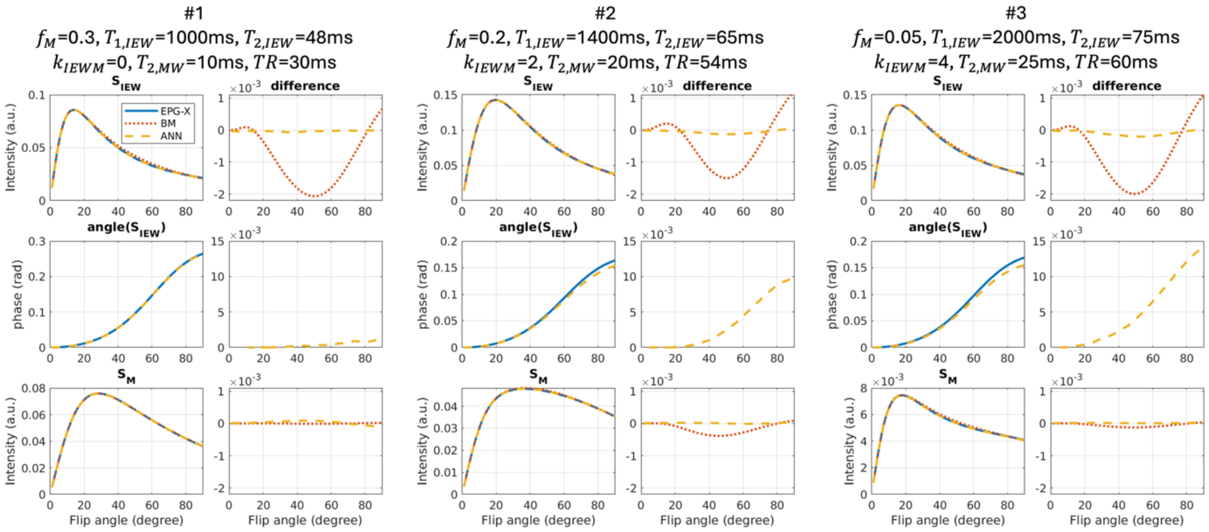

Figure S2: Steady-state signals derived from EPG-X, Bloch-McConnell equation, and ANN in three representative parameter sets. Comparisons highlight the reduced bias of ANN relative to BM, particularly for the IEW compartment.
